# Microglial Innate Immune Memory: Implications and Research Advances in Central Nervous System Disorders

**DOI:** 10.3390/cimb48040426

**Published:** 2026-04-21

**Authors:** Yaru Song, Shiyi Shu, Xiansi Zeng, Manli Xia, Junru Liu, Li Li

**Affiliations:** 1 College of Life Science and Medicine, Zhejiang Sci-Tech University, Hangzhou 310018, China; 2023220902037@mails.zstu.edu.cn (Y.S.); 2024220902042@mails.zstu.edu.cn (S.S.); 2 Medical College, Jiaxing University, Jiaxing 314000, China; xszeng@zjxu.edu.cn (X.Z.); xiamanli@zjxu.edu.cn (M.X.)

**Keywords:** microglia, immune training, immune tolerance, signal pathway, central nervous system

## Abstract

The central nervous system (CNS), comprising the brain and spinal cord, represents the core regulatory hub of the body. Damage to the CNS often leads to irreversible structural and functional impairments of neural tissues, posing a major global public health challenge. Immune memory encompasses two states: immune training and immune tolerance, which are characterized by enhanced or attenuated immune responses, respectively, following initial exposure to external stimuli in immune cells such as monocytes and macrophages. Microglia, the resident immune cells of the CNS, can be rapidly activated by external stimuli. Accumulating evidence indicates that microglial immune memory plays a critical role in sustaining states and neuroinflammatory responses in CNS disorders. Specifically, the immune training state promotes amyloid-β (Aβ) accumulation in the brains of Alzheimer’s disease (AD) model mice, thereby exacerbating neuronal damage, whereas the immune tolerance state suppresses pro-inflammatory cytokine expression and alleviates neuroinflammation. This review focuses on two immune memory states in microglia—training and tolerance—and what triggers them. We summarize their roles and mechanisms in CNS diseases. Specifically, we break down how epigenetic and metabolic reprogramming control microglial immune memory, with an emphasis on how these two processes interact during memory formation and maintenance. Our goal is to fill key knowledge gaps about their combined effects and to suggest new therapeutic targets. Evidence shows that immune memory acts as a “double-edged sword” in the CNS: it can either fuel harmful inflammation and worsen damage, or, when moderately activated, protect nerves. Therefore, precisely balancing these two states could help reduce harmful inflammation while preserving the protective functions of microglia, offering a new, reversible immunotherapy for CNS diseases.

## 1. Introduction

In the last century, the central nervous system (CNS) was thought to lack lymphatic vessels with immune surveillance functions and specialized antigen-presenting cells, leading to its characterization as an immune-privileged or immune-isolated system [1,2,3]. However, this traditional view has been completely overturned by subsequent research. The CNS is now recognized as a dynamic immune environment, primarily due to the dominance of microglia and other resident macrophages [4]. Microglia are innate immune cells in the CNS, originating from the extraembryonic yolk sac [5,6]. They permanently reside in the brain parenchyma and maintain their population through self-renewal, which is essential for sustaining nervous system homeostasis [6]. Conventionally, it was believed that only adaptive immune systems possess antigen-specific memory cells (T cells and B cells) capable of mediating long-term immune protection, whereas the innate immune system lacks such memory characteristics [7]. However, with advancing research in CNS immunology, this perspective has been gradually overturned. Microglia, as classical resident innate immune cells in the CNS, not only serve as the first line of innate immune defense against external stimuli but have also been demonstrated to possess unique innate immune memory capabilities [8]. In addition, they also possess unique innate immune memory (IIM) capabilities [9]. Following initial exposure to external stimuli, microglia undergo persistent alterations in phenotypic, functional, or reactive states. When re-exposed to the same or different stimuli, their immune responses may be either enhanced or attenuated. This phenomenon is collectively termed IIM, which encompasses two opposing states: innate immune training (TR) and innate immune tolerance (TL) [10].

The formation of IIM in microglia is driven by both metabolic and epigenetic reprogramming. Following stimulation by lipopolysaccharide (LPS), microglial retain memory prior inflammatory activation states within their cellular enhancer regions. Persistent epigenetic modifications in the enhancer regions contribute to the establishment of long-term cellular memory [11,12]. Upon initial stimulation, histone H3K9/K14 acetylation and H3K4 methylation at the IL-1β promoter are activated to initiate transcription; however, subsequent LPS stimulation suppresses its transcriptional levels [13]. The transition from immune training to immune tolerance is often accompanied by shifts in metabolic status. For instance, in mouse models of Alzheimer’s disease (AD), microglia under immune training exhibit upregulated aerobic glycolysis, which promotes the enrichment of H3K4me1 and H3K27ac at IL-1β enhancer regions, ultimately exacerbating amyloid-β (Aβ) deposition. In contrast, microglia in an immune tolerance state display inhibited glycolysis, restored oxidative phosphorylation and suppression of the epigenetic modifications observed during training, thereby significantly mitigating AD pathological progression [9]. Collectively, changes in metabolic state and epigenetic modifications at the molecular level jointly promote the formation of IIM in microglia.

In CNS diseases, the IIM of microglia exerts bidirectional regulatory effects. Depending on the frequency and duration of external stimuli, microglial can be induced into either an immune training or tolerance state, which produces opposing disease outcomes. For example, in mouse models of AD, a single intraperitoneal injection of LPS led to significantly increased Aβ plaque load and total Aβ protein levels in the brain six months post-injection, accompanied by impaired microglial Aβ clearance, thereby exacerbating AD pathology. In contrast, four consecutive intraperitoneal LPS injections over six months resulted in significant reductions in Aβ plaque load and total Aβ protein levels, along with markedly suppressed pro-inflammatory responses, demonstrating neuroprotective effects [9]. Furthermore, the innate immune memory of microglia has been implicated in the pathogenesis and progression of various CNS disorders, including Parkinson’s disease, multiple sclerosis, psychiatric disorders, and brain injuries [14,15,16]. Thus, its regulatory mechanisms may serve as potential targets for therapeutic intervention.

Since the concept of microglial innate immune memory was first proposed in 2018 [9], an increasing number of studies have investigated its role in various diseases over the past decade. Nevertheless, several key questions remain unresolved: whether immune tolerance can be reversed to an immune-trained state, whether immune memory exerts consistent effects across different brain regions, whether immune cells other than glial cells also possess immune memory capacity, and whether metabolic inhibitors or epigenetic modulators can be translated into clinical trials for CNS diseases. This review focuses on the core concept of microglia IIM, summarizes the characteristics of its two states, discusses current research and underlying mechanisms in CNS diseases, and outlines future research directions. Our aim is to provide novel insights for the therapeutic application of microglia IIM in CNS disorders.

## 2. Microglia

Microglia are mononuclear macrophages widely distributed throughout the brain and spinal cord, accounting for approximately 5–20% of the total cell population in various brain regions of mice [17] and 0.5–16.6% in different brain regions of humans [18]. The roles of microglia in the CNS are highly complex, exhibiting both neuroprotective and neurotoxic effects [19]. Under normal physiological conditions, microglia play a crucial role in neuroprotection by phagocytosing cellular debris and damaged neurons [20,21], as well as by receiving and releasing various signaling molecules [22]. They are closely associated with neuronal cells [23]. Upon injury stimulation, rapid microglial activation induces quantitative and morphological changes and can also lead to neuronal damage and neuroinflammation through the release of various pro-inflammatory cytokines and mediators [24,25]. However, when stimulation persists, epigenetic modifications occur in enhancer regions within microglia, primarily establishing and preserving immune memory for the previous inflammatory activation state. These epigenetic alterations exhibit stability and durability, thereby conferring immune memory functionality to microglia [26,27]. This challenges the traditional theory that only adaptive immunity can generate immune memory, demonstrating that innate immunity also possesses the capacity to form long-term and stable immune memory.

## 3. Immune Memory

Immune memory has been documented in the history of human infectious diseases. Records of the Athens plague indicate that individuals who had recovered from plague infection exhibited reduced susceptibility upon re-exposure to the same pathogen, confirming that repeated infections can induce protective effects associated with the adaptive characteristics of T cells and B cells [28]. However, studies over the past decade have confirmed that innate immune cells also undergo sustained functional reprogramming following initial stimulation, exhibiting either enhanced or attenuated immune responses upon secondary challenge. Microglia, which mediate immune responses to external stimuli, serve as key carriers of immune memory in the CNS. Intraperitoneal injection of poly(I:C) during mouse pregnancy induces strong maternal innate immune responses, leading to increased release of pro-inflammatory factors and affecting microglial development and function in offspring brains. Adult offspring exposed to this immune stimulation maintain persistently activated microglia, with morphological features (branched morphology and enlarged cell bodies) favoring an activated state. Their phagocytic capacity for clearing debris and apoptotic cells is significantly impaired, accompanied by cognitive dysfunction. These findings suggest that microglia in offspring brains may retain long-term memory of inflammatory stimuli encountered during maternal pregnancy [29,30]. In addition, microglia in the brains of adult mice also undergo morphological changes following stimulation. Four weeks after tail vein injection of attenuated Salmonella typhimurium, mice received a low-dose LPS challenge (which does not induce inflammatory responses in naive mice) as a second stimulus. The results demonstrated that microglia exhibited stronger and more rapid hyperreactivity, leading to cerebral vascular endothelial dysfunction and impaired tight junction proteins [31]. Notably, Wendeln et al. were the first to directly demonstrate in vivo that single or repeated intraperitoneal injections of LPS induce epigenetic reprogramming in hippocampal microglia for up to six months, thereby establishing stable IIM [9].

## 4. Two States of IIM in Microglia

Depending on the duration and frequency of exogenous stimuli (e.g., inflammation or pathogens), microglial IIM is generally classified into two opposing states: immune training and immune tolerance. Immune training refers to an enhanced immune response upon re-exposure to a second stimulus, whereas immune tolerance denotes a suppressed immune response under the same condition [9,32]. Two states are typically distinguished by evaluating the expression levels of pro-inflammatory and anti-inflammatory factors. Following peripheral immune stimulation, microglia exhibited altered levels of epigenetic modification, which concurrently influenced transcriptional and functional changes. The specific characteristics of these two immune memory states are summarized in Table 1.

### 4.1. Immune Training Status

Immune training refers to sustained functional changes in microglia, mediated by epigenetic and metabolic reprogramming following initial immune stimulation, that enable a stronger inflammatory response upon re-exposure to the same or a novel stimulus [10,33]. The induction of immune training involves external stimuli that trigger epigenetic modifications in microglia, thereby altering the transcriptional expression of pro-inflammatory genes. In normal and AD model mice, LPS was used to induce immune training in brain microglia at three months of age. At nine months, microglia were isolated from the hippocampus and subjected to chromatin immunoprecipitation sequencing for H3K4me and H3K27me. The results confirmed that the elevation of H3K4me levels was primarily induced by LPS, with enhancers predominantly enriched in inflammation-related pathways such as mTOR-HIF-1α, indicating the onset of inflammatory responses [9]. In another study, two intraperitoneal injections of LPS in two-month-old mice induced microglial into an immune-trained state, characterized by significantly elevated expression of pro-inflammatory factors at both protein and mRNA levels, peak inflammatory responses, weight loss and microglial activation [34]. Another mechanism involves changes in metabolic status. Following immune training, intracellular glycolysis is enhanced, providing a fundamental metabolic basis for cellular functions [35]. In the BV-2 microglial cell line, immune training induction led to a marked increase in aerobic glycolysis and the expression of glycolysis-related genes. Moreover, inhibition of glycolysis using sodium oxalate prevented the establishment of immune-trained microglial cells [36]. Immunometabolomics has revealed that microglia in AD undergo substantial metabolic alterations during disease progression due to dynamic functional and phenotypic changes. Recent studies have shown that Aβ plaques and tau tangles can induce immune training in microglia, leading to markedly increased expression of key glycolytic enzymes and activation of the HIF-1α signaling pathway. This enhances glycolytic metabolism and subsequently promotes the secretion of pro-inflammatory factors, thereby exacerbating neuroinflammation [37]. Collectively, these findings indicate that metabolic status and epigenetic modifications are critical processes driving the generation of long-term immune memory in microglia.

### 4.2. Immune Tolerance State

Immune tolerance is a memory state in which innate immune cells undergo functional reprogramming following an initial stimulus, resulting in attenuated inflammatory responses upon subsequent immune stimuli [35]. Following prolonged low-dose endotoxin stimulation, these cells exhibit reduced release of pro-inflammatory factors and increased expression of anti-inflammatory factors, a phenomenon initially observed in peripheral monocytes and macrophages [36]. Recent studies have confirmed that microglia in the CNS can also establish immune tolerance [38]. Wendeln et al. administered four LPS injections to three-month-old AD model mice. Six months later, they observed a significant reduction in Aβ plaque load, and the expression of the pro-inflammatory cytokine IL-1β decreased to levels comparable to those in wild-type mice [9]. To validate the role of immune memory in other disease contexts, researchers established a focal cerebral ischemia model one month after four LPS injections. By day 7 post-ischemia, neurological damage showed a recovery trend, accompanied by a significant reduction in microglial activation volume [9]. Schaafsma et al. further demonstrated that immune tolerance modulates inflammatory cytokine expression, specifically manifested as elevated levels of the repressive histone modification H3K9me2 in the IL-1β promoter region following two rounds of LPS-induced immune tolerance. In addition to suppressing pro-inflammatory responses, microglia exhibited enhanced phagocytic activity, increased outward potassium currents, and elevated nitric oxide production during the second LPS challenge [39]. Beyond alterations in H3K9 modification levels, microglia sorted from the hippocampus of immune-tolerant mice showed significantly elevated H3K4 modification levels in Rap1 signaling pathway molecules and in putative enhancers associated with phagocytic function [9]. Moreover, when the number of LPS injections was increased to four, microglia transitioned from an immune-trained to an immune-tolerant state. Transcriptomic analysis revealed marked suppression of the pro-inflammatory NF-κB pathway and significant upregulation of anti-inflammatory and oxidative phosphonate pathways [34]. Collectively, these findings are consistent with previous reports demonstrating that immune tolerance reduces inflammatory gene expression through epigenetic reprogramming, enhances microglial function, and ameliorates disease progression.

**Table 1:** Comparison of characteristics of microglia immune training and immune tolerance.

Feature Category	Innate Immune Training	Innate Immune Tolerance	Refs
Definition	After the first stimulation, an enhanced immune response is produced to the second homologous or heterologous stimulation	After the first stimulation, the immune response to the second homologous or heterologous stimulation is weakened	[9,40]
Typical triggering factors	LPS (1×LPS) β-glucan (Short-term stimulation)	LPS (4×LPS) β-glucan (Long-term stimulation)	[34,41]
Metabolic reprogramming	Glycolysis ↓	Oxidative phosphorylation ↑	[42,43]
Epigenetic changes	H3K4me1/3, H3K27ac, H3K18la	H3K9me2/3, H3K27me3	[33,44]
Expression of inflammatory	Pro-inflammatory factors increased, anti-inflammatory factors decreased	Pro-inflammatory factors decreased, anti-inflammatory factors increased	[9]
Main functional effect	Aggravate disease pathology	Relieve disease pathology	[9]
The role in CNS diseases	Aggravate neuroinflammatory response Increase Aβ deposition Neuronal damage	Play a neuroprotective role Appropriate activation of microglia Reduce neuroinflammatory response	[9,15]
Biomarker	LDHA, HIF-1α TBF-α, IL-1β, IL-6 IBA-1, CD68	PDH, SDH, IDH2 IL-10, TGF-β, Arg-1 CD206	[15,45,46,47]
Clinical significance	Anti-infective, anti-tumor, exacerbate inflammation, autoimmune	Improving cardiovascular disease, promoting tumor immune evasion	[9,48,49,50]

## 5. Inducer of Immune Memory

The generation of innate immune memory in microglia requires specific inducers, which can be either endogenous (e.g., interleukins, interferons) or exogenous substances (e.g., LPS, β-glucan). These inducers bind to corresponding receptors on microglial cells, activate downstream signaling pathways, and subsequently alter cellular morphology and activation state. Through epigenetic reprogramming and metabolic modifications, microglia develop memory responses to stimuli.

### 5.1. LPS

LPS is the primary component of the outer membrane of Gram-negative bacteria and a commonly used inflammatory inducer [51]. As a potent pro-inflammatory agent, it has been widely employed in both in vitro and in vivo mouse models to establish sepsis and inflammatory conditions. LPS is recognized by the pattern recognition receptor TLR4, which mediates the activation of NF-κB and MAPK signaling pathways. This leads to increased transcription of pro-inflammatory factors, enhances glycolysis and oxidative phosphorylation, and subsequently epigenetic modifications (e.g., methylation and acetylation), ultimately regulating downstream gene expression [52,53,54]. Recent studies have shown that LPS can also induce innate immune memory in microglia, a finding validated in various mouse disease models. Adult mice received intraperitoneal injections of low-dose LPS (0.5 mg/kg) for four consecutive days to assess microglial immune memory across different brain regions. Pro-inflammatory factors (IL-1β and TNF-α) peaked in the prefrontal cortex and hippocampus two days post-injection, indicating the establishment of an immune-trained state. These biomarkers returned to baseline levels four days after injection, suggesting a transition toward immune-tolerant status [9]. In mouse models of Parkinson’s disease (PD), a single LPS injection exacerbates neuronal damage and inflammatory responses, increasing the proportion of pro-inflammatory microglia; in contrast, repeated injections alleviate these pathological changes and elevate the proportion of anti-inflammatory microglia. LPS dose also determines the immune memory phenotype: low-dose LPS induces immune training (enhanced pro-inflammatory factor expression), whereas high-dose LPS induces immune tolerance (reduced pro-inflammatory factors and increased anti-inflammatory factor release) [15]. Beyond LPS alone, combinations with other agents can further potentiate immune memory induction. For example, in the human-derived microglial cell line HMO6, cells were first treated with LPS and water-soluble chitosan for 24 h, rested for 24 h, and then re-exposed. This regimen resulted in significantly higher iNOS expression compared to LPS alone, indicating that microglia remained in a sustained immune-trained state. Thus, water-soluble chitosan enhances microglial responsiveness to subsequent LPS exposure [43]. Similarly, combined exposure to LPS and manganese (Mn)—an environmental toxin associated with PD—alters epigenetic reprogramming. Administration of Mn (1 μg/mL) for 3 h following LPS induction activates the NLRP3 inflammasome pathway, with pro-inflammatory responses peaking at 13 h and gradually returning to homeostasis after 48 h [44]. LPS has now become a classical tool for inducing IIM in microglia. By controlling dosage and stimulation frequency, LPS can drive microglia toward opposite IIM phenotypes.

### 5.2. β-Glucan

β-glucan, a natural polysaccharide found in fungal cell walls, exhibits potent immunomodulatory effects and is the earliest and most widely used in vitro inducer of innate immune memory [55]. It initiates signal transduction primarily by binding to pattern recognition receptors (e.g., Dectin-1 and TLR4) on the microglial surfaces [56,57], subsequently inducing metabolic reprogramming and epigenetic remodeling that lead to the formation of a stable innate immune memory phenotype. This induced memory phenotype is strongly pro-inflammatory, directing microglia toward an immune-trained state [57]. Mechanistically, β-glucan stimulation triggers marked metabolic reprogramming, characterized by robust activation of glycolytic enzymes including hexokinase 2 (HK2) and lactate dehydrogenase A (LDHA), which are upregulated, resulting in substantial lactate accumulation [58]. Concurrently, significant epigenetic remodeling occurred, with the promoter region of the pro-inflammatory gene (IL-1β) remaining in an open state [59]. Together, these changes induce a long-term, hyperreactive pro-inflammatory memory in microglia.

In CNS disorders, β-glucan-induced immune training memory plays a crucial regulatory role in disease pathogenesis. Studies in mouse models of depression-like behavior have shown that β-glucan activates microglia via the Dectin-1-mediated SrcSyk tyrosine kinase pathway, reversing chronic stress-induced reduction in hippocampal microglial numbers and functional impairment. In cultured microglia, β-glucan induced reactive oxygen species (ROS) production without significantly upregulating classical pro-inflammatory cytokines such as IL-1β, IL-6, or TNF-α, thereby avoiding the neuroinflammatory side effects associated with conventional stimulants [60]. In a study of AD mice, β-glucan administration reduced microglial activation, diminished chronic neuroinflammation, delayed Aβ plaques deposition, and ultimately improved cognitive function [61].

Current research has elucidated the core regulatory mechanisms and disease associations of β-glucan as a modulator of microglial IIM. However, numerous unexplored directions remain, including the cell-specific regulatory network underlying β-glucan-induced microglial immune memory, its synergistic interactions with other modulators, and differential regulatory effects across various CNS disorders. Addressing these questions will further our understanding of the induction mechanisms of microglial IIM and provide critical support for developing novel intervention strategies targeting CNS diseases through modulation of microglial immune memory.

### 5.3. Cytokine

Inflammatory factors drive changes in microglial phenotypes by binding to receptors and activating inflammation-related signaling pathways, serving as key upstream regulators of microglial activation states and functional phenotypes. Interferon-gamma (IFN-γ) is a critical mediator of microglial activation and participates in immune priming and immune training processes. The activation of microglia toward a pro-inflammatory phenotype requires the synergistic action of IFN-γ and the TLR4 signaling pathway. This process induces the synthesis of inducible iNOS and promotes the secretion of pro-inflammatory cytokines, while simultaneously reducing neurotrophic factors release, thereby triggering inflammatory responses [62,63]. This mechanism resembles the phenotypic changes observed in microglia after four intraperitoneal injections of LPS, suggesting that IFN-γ may be associated with microglial immune memory. Transforming growth factor-β1 (TGF-β1) is a key factor in maintaining microglial homeostasis [64]. Transcriptomic analysis of microglia isolated from a mouse model of amyotrophic lateral sclerosis (ALS) revealed downregulated expression levels of Tgfbr1 and Tgfb1 genes, leading to phenotypic alterations in microglia [65]. Although cytokines primarily influence microglial gene expression through transcriptional regulation, post-transcriptional regulation mediated by their oversecretion also plays a significant role.

### 5.4. Pathological Protein

Alpha-synuclein (α-Syn) is a pathologically relevant protein in PD. Excessive deposition of α-Syn in neurons and glial cells leads to the formation of Lewy bodies and Lewy neurites, which disrupt cellular function and result in neuronal death and progressive cognitive decline [66,67]. Primary microglia were treated with PD-derived α-Syn and LPS (as a control). After 48 h, microglia in the LPS-treated group transformed from a resting branched morphology to an activated ameboid morphology. Similar morphological changes were observed in the α-Syn-treated group, indicating that α-Syn can also trigger inflammatory responses [66]. α-Syn induces activated microglia to release IL-6, which promotes neuronal iron uptake and reduces intracellular iron efflux, leading to neurotoxic iron accumulation in neurons [68]. Additionally, α-Syn stimulates microglial activation and IL-1β secretion the NLRP3 inflammasomes [69]. During this process, NLRP3 activation leads to caspase-1 cleavage and activation, which subsequently cleaves and promotes and secretes IL-1β [70]. Thus, NLRP3 can be considered a key mediator in α-Syn-induced microglial memory.

## 6. Molecular Mechanisms of IIM

The generation of IIM is highly dependent on epigenetic reprogramming, a process that involves not merely alterations in gene expression but rather the reshaping of cellular fate through epigenetic modifications [71]. Epigenetic reprogramming primarily includes DNA methylation, post-translational covalent modifications of histones, and acetylation modifications [72]. In addition to epigenetic reprogramming, alterations in cellular metabolic pathways also participate in the regulation of immune memory. Following external stimuli, the balance between glycolysis and oxidative phosphorylation in cells undergoes dynamic changes [73]. This, in turn, affects the activity of enzymes involved in energy supply and metabolite processing, ultimately influencing the efficacy of cellular immune memory [74].

### 6.1. Epigenetic Reprogramming

In microglia, epigenetic reprogramming is typically achieved through epigenetic modifications occurring in cellular enhancer regions [75]. Enhancers are cis-regulatory elements in the genome that can remotely regulate the transcriptional activity of target genes, a process highly dependent on the dynamic balance of epigenetic modifications acting on histones at the post-translational level. Several histone modifications exhibit strong plasticity in microglial innate immune memory. The most extensively studied is histone acetylation modification. Using Mn as the initial stimulus for microglia, specific deposition of H3K27ac at microglial enhancers was observed following secondary LPS stimulation, exacerbating neuroinflammatory responses. However, pharmacological inhibition of GNE-049 suppressed the formation of epigenetic memory, thereby inhibiting iNOS transcription and the activation of pro-inflammatory pathways [44]. Lead poisoning induces neuroinflammation and exacerbates learning and memory impairments. Treatment with sodium butyrate inhibits ATAT3 signal activation in microglia, marks enhancer regions to activate H3K9ac transcription, and enhances the expression of neuroprotective factors such as Brain-Derived Neurotrophic Factor (BDNF) [76]. In a mouse model of IIM, isolated microglia exhibited suppressed IL-1β expression. Although H3K9/K14ac at the IL1B promoter was activated following initial stimulation, transcription was inhibited upon re-exposure to LPS [9]. These findings indicate that enhancer regions retain memory of prior inflammatory activation states, and that alterations in epigenetic modifications within these regions contribute to the generation of long-term cellular memory. Histone methylation also plays a pivotal role. In a PD mouse model in which Mn pretreatment followed by secondary LPS stimulation induces immune memory, elevated expression levels of H3K4me3 and H3K4me1 were observed in the substantia nigra, striatum, and olfactory bulb. This aligns with clinical studies demonstrating increased H3K4me3 deposition in the brains of PD patients. However, while H3K4me1 expression showed an upward trend, the increase remained statistically insignificant [44]. These results suggest that H3K4me3 and H3K4me1 may serve as epigenetic markers for immune regulation. In studies investigating the role of kynurenine acid (KYNA) in brain anti-inflammatory responses, KYNA was found to reduce the expression of the inflammation-related proteins CXCL10 and CCR1 in primary microglia following LPS treatment, accompanied by increased H3 deposition in the nucleus. However, the expression patterns of H3K9me2 and H3K9me3 exhibited distinct trends. Immunofluorescence co-localization analysis revealed abnormal intracellular distribution of H3K9me3, with its migration from the nucleus to the cytoplasm, whereas H3K36me2 showed no significant intracellular movement, transitioning from the nucleus to the extranuclear space. These heterochromatin markers exert inhibitory effects on pro-inflammatory gene expression. The nuclear-to-cytoplasmic shift of H3K9me3 attenuates nuclear gene repression, thereby enhancing inflammatory responses [77]. This indicates that nuclear-to-protoplasmic shuttling of H3K9me3 is a critical epigenetic modification phenomenon in microglial inflammatory activation.

### 6.2. Metabolic Reprogramming

Epigenetic reprogramming regulates the generation of immune memory in innate immune cells and serves as the molecular mechanism that modulates the enhancement or attenuation of immune responses upon secondary stimulation. Stimulation by IIM inducers leads to epigenetic modifications that cause substantial gene accumulation in promoter regions, resulting in alterations in chromatin transcriptional activity and inducing changes in cellular metabolic states [78]. Thus, a complex and close relationship exists between metabolic reprogramming and epigenetic modifications, which jointly regulate the formation of IIM. IIM has been demonstrated to play a role in pathways such as glycolysis, oxidative phosphorylation, tricarboxylic acid cycle, and lipid metabolism [79]. During innate immune training in microglia, the glycolytic pathway is enhanced, accompanied by upregulation of key glycolytic enzymes, accumulation of TCA cycle intermediates, and increased production of lactate, which is considered a metabolic waste product. In contrast, during immune tolerance, the cellular metabolic state shifts predominantly toward oxidative phosphorylation, with glycolysis and its associated enzymes exhibiting suppressed expression [80]. These metabolic alterations are relevant to CNS disorders and may correlate with microglial activation states [81]. When microglia are in an activated state induced by immune training, they require substantial substrates to provide energy for subsequent immune response enhancement. When microglia transition to immune tolerance, the cells remain in a resting state, resulting in lower energy demands [35]. However, in AD, the progressive accumulation of pathological Aβ protein may induce an immune-tolerant state in microglia. During this phase, both glycolytic and oxidative phosphorylation metabolic pathways are downregulated, impairing microglial function and thereby exacerbating AD pathology [37]. Microglia with immune memory and enriched in *APOE4* genes exhibit a disordered TCA cycle, a metabolic bias toward glycolysis, and significant accumulation of intermediates such as lactate and succinate following inflammatory induction—characteristics typical of immune training [82]. In addition to glucose metabolism, increased expression of several key enzymes in the cholesterol synthesis pathway is also critical for inducing IIM. For example, ACSL4 promotes microglia-mediated neuroinflammation by regulating lipid metabolism; ACSL4 catalyzes the formation of acyl-CoA from polyunsaturated fatty acids such as arachidonic acid. This process facilitates phospholipid re-synthesis and the synthesis of pro-inflammatory lipid mediators, thereby remodeling the pro-inflammatory metabolic phenotype of microglia [83]. Downregulation of ACSL4 can significantly reverse this metabolic process, inhibit the transformation of microglia into a pro-inflammatory phenotype, and provide therapeutic targets for treating neurodegenerative diseases by modulating IIM.

Metabolic reprogramming serves as the core driving force for inducing dynamic alterations in epigenetic modifications of microglia. These two processes are not independently regulated but rather achieve a multidimensional synergistic effect through metabolic product supply, signaling pathway mediation, and chromatin accessibility shaping, thereby facilitating the transition from metabolic states to epigenetic characteristics and ultimately establishing the immune memory phenotype of microglia [35,84,85]. Metabolic processes can directly regulate the activity of epigenetic modification enzymes through their products. Under immune training conditions, aerobic glycolysis in microglia is significantly enhanced, leading to the accumulation of intracellular metabolites such as acetyl-CoA and pyruvate. These metabolites not only provide sufficient substrates for histone acetyltransferases (HATs), promoting the establishment of active histone acetylation modifications such as H3K27ac and H3K9/K14ac, but also activate histone methyltransferases. This process facilitates the enrichment of pro-inflammatory-related methylation modifications in inflammatory gene regulatory regions, keeping chromatin in an open state to enhance the transcription of pro-inflammatory genes [53]. Under immune tolerance conditions, glycolysis is inhibited and oxidative phosphorylation is restored. The expression of itaconate increases, an organic acid in the TCA cycle, which can inhibit succinate dehydrogenase. It also synergizes with lactate to enhance inhibitory epigenetic modifications, leading to chromatin condensation in pro-inflammatory gene regions and thereby suppressing their transcriptional expression [86]. Meanwhile, core signaling molecules in metabolic pathways can indirectly regulate epigenetic modifications. For instance, the mTOR-HIF-1α pathway activated during immune training leads to nuclear translocation of the downstream transcription factor HIF-1α, which enhances glycolysis and results in accumulation of metabolites such as lactate, fumarate, and NAD+. These metabolites inhibit histone demethylase activity, elevate H3K4me3 epigenetic modifications in the promoter regions of pro-inflammatory genes (e.g., TNF-α and IL-6), and place chromatin in a transcriptionally active state [87]. The suppressed NF-κB pathway and moderately activated PI3K-AKT pathway in immune tolerance downregulate the expression of pro-inflammatory-associated epigenetic modification enzymes while upregulating the expression of inhibitory epigenetic modification enzymes, thereby further guiding the formation of inhibitory epigenetic signatures [88].

## 7. Signaling Pathways Related to IIM

**Figure 1:**
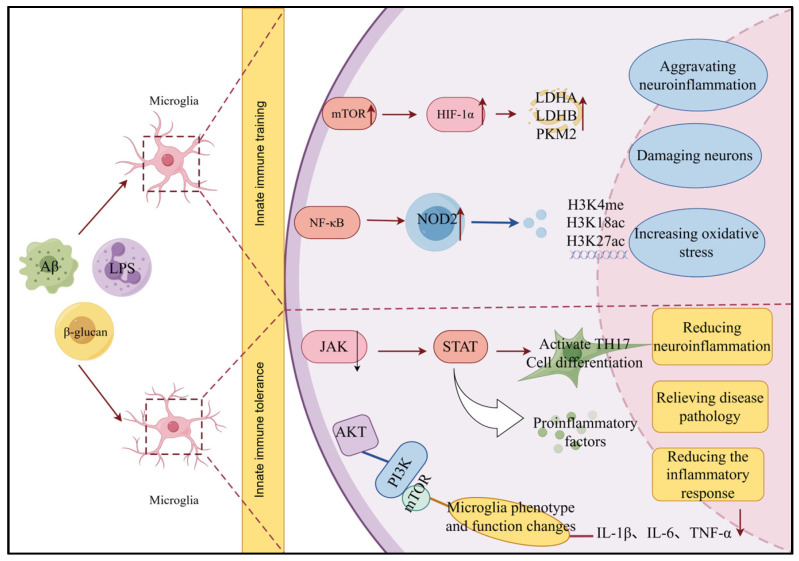
Diagram of signaling pathways related to microglial innate immune memory. Signaling pathway alterations in microglia during immune training and immune tolerance under distinct immune stimuli, including Aβ, LPS and β-glucan. In the immune training state, enhanced glycolysis and epigenetic modifications including H3K4me and H3K18ac promote hyper-responsive pro-inflammatory activity. Conversely, in immune tolerance, microglia attenuate neuroinflammation and immune injury via metabolic reprogramming and suppression of pro-inflammatory signaling. Key regulatory pathways including mTOR-HIF-1α, NF-κB-NOD2 and JAK-STAT are highlighted, illustrating their roles in modulating microglial function and neuroprotective outcomes (The image was drawn on Figdraw; https://www.figdraw.com/static/index.html#/; accessed on 19 December 2025). Aβ: Amyloid β-protein, LPS: lipopolysaccharide, mTOR: Mammalian target of rapamycin, HIF-1α: Hypoxia inducible factor-1, LDHA: Lactate dehydrogenase A, LDHB: Lactate dehydrogenase B, PKM2: Pyruvate Kinase M2, NF-κB: Nuclear factor-κB, NOD2: Nucleotide-binding oligomerization domain 2, JAK: Janus kinase, STAT: Signal transducer and activator of transcription, PI3K: Phosphatidylinositol-3 kinase, IL-1β: Interleukin-1 beta, IL-6: Interleukin-6, TNF-α: Tumor necrosis factor-alpha. Upward arrow indicates increased expression, and a downward arrow indicates decreased expression.

After inducing innate immune memory, microglia immediately mount immune responses to subsequent identical or different stimuli, activating multiple signaling pathways, leading to the secretion of large amounts of inflammatory factors, and influencing cell proliferation, phenotype, and function. Recent studies have found that the pathways associated with innate immune memory share certain similarities with inflammatory pathways, metabolic pathways, and others. Figure 1 summarizes the signaling pathways related to innate immune memory.

### 7.1. mTOR Signaling Pathway

The mTOR-HIF-1α signaling pathway plays a key role in aerobic glycolysis by enhancing the metabolic capacity of immune cells to support the persistence of immune memory [89]. Cheng et al. demonstrated that immune training not only relies on epigenetic modifications but is also associated with metabolic reprogramming, particularly during aerobic glycolysis. mTOR and HIF-1α serve as key regulatory factors for glycolytic gene expression. mTOR drives the expression of glycolysis-related genes by activating HIF-1α, thereby promoting metabolic transition. Inhibition of this pathway attenuates immune responses [46]. Under Aβ stimulation, the expression of mTOR and HIF-1α proteins is significantly upregulated, and glycolysis-related genes are transcribed, indicating activation of this pathway and a shift in microglia from oxidative phosphorylation to aerobic glycolysis. During the immune tolerance phase, inactivation of this pathway leads to loss of immune memory and metabolic defects in microglia. However, when IFN-γ reactivates the mTOR-HIF-1α pathway, glycolysis levels are restored and the phagocytic and Aβ clearance functions of microglia are improved [90]. In the brain tissue of AD mice, IFN-γ expression levels were relatively low. Targeting Tregs to enhance IFN-γ expression could break the microglial immune tolerance state induced by OAβ. This intervention was found to increase AKT-mTOR expression levels, promote glycolysis, and exacerbate inflammatory responses [91]. In addition to IFN-γ improving disease outcomes by altering immune memory status through the AKT-mTOR signaling pathway, certain drug-loaded nanoparticles can also induce microglial polarization from pro-inflammatory to anti-inflammatory by blocking the Akt-mTOR-HIF-1α signaling pathway, thereby restoring cerebral energy metabolism. After being encapsulated by nanomaterials and delivered into microglial cells, these nanoparticles inhibit AKT phosphorylation and reduce mTOR and HIF-1α expression levels, consequently suppressing glycolytic gene transcription and facilitating a metabolic shift from glycolysis to OXPHOS [92].

### 7.2. PI3K-AKT Signaling Pathway

AKT signaling plays a pivotal role in regulating cellular activities under both pathological and pathological conditions. As one of the most common signaling cascades in cells, it is extensively involved in the pathogenesis and progression of inflammatory responses, immune dysregulation, and cancer development [93,94]. The PI3K-AKT signaling pathway serves as a core regulatory hub for microglia in establishing and maintaining pro-inflammatory innate immune memory. Studies have demonstrated that the TREM2 receptor, by coupling with the PI3K-AKT signaling pathway, not only mediates the phagocytic function of microglia but also participates in the transformational memory of their functional states. Once this signaling pathway becomes dysregulated, microglia will transition to a pro-inflammatory phenotype and accelerate neurodegenerative processes [95]. Furthermore, the failure of the negative feedback mechanism within the PI3K-AKT pathway leads to persistent alterations in microglial sensitivity to inflammatory stimuli, thereby driving chronic neuroinflammatory processes in diseases such as AD and multiple sclerosis [96]. Intervention strategies targeting this pathway have demonstrated potential in modulating immune memory. For example, natural products can reverse the “inflammatory memory” of microglia by activating the PI3K-AKT pathway, promoting their transition to an anti-inflammatory phenotype, thereby offering novel therapeutic approaches for CNS disorders [97]. As a representative example, the natural flavonoid quercetin drives the phenotypic conversion of pro-inflammatory microglia toward an anti-inflammatory state by upregulating p-PI3K/p-AKT and inhibiting downstream NF-κB. The complete reversal of this effect by the PI3K inhibitor LY294002 confirms that this pathway represents a critical entry point for reversing pathological immune memory. Thus, targeting the PI3K-AKT signaling axis may constitute an effective strategy for correcting aberrant microglial immune memory and treating CNS disorders [98]. In experiments using different doses of LPS to induce innate immune memory in microglia, it was found that the LPS stimulation dose can determine the immune memory phenotype of microglia through the PI3Kγ-AKT axis. Ultra-low doses of LPS induce immune training by activating the PI3Kγ-AKT-mTOR/HIF-1α pathway, enhancing glycolysis and promoting the massive release of pro-inflammatory factors such as TNF-α and IL-6 during secondary stimulation, thereby forming inflammatory memory. In contrast, high doses of LPS inhibit the PI3Kγ-AKT signaling pathway, reduce glycolysis, and upregulate IL-10, mediating immune tolerance. PI3Kγ knockout or kinase inactivation completely blocks LPS-induced AKT activation and immune memory formation, indicating its role as a key regulatory molecule [42].

### 7.3. NF-κB Signaling Pathway

The NF-κB transcription factor family plays critical roles in various cellular functions, including inflammatory responses, apoptosis, cell survival, proliferation, angiogenesis, as well as innate and adaptive immunity [99]. In pharmacological studies of LPS-induced neuroinflammation, pretreatment with gastrodin was found to ameliorate LPS-induced neuroinflammatory responses, reduce the expression of proteins in the TLR4/TRAF6/NF-κB signaling pathway and Stat3 phosphorylation, and induce microglial cells to transition from pro-inflammatory to anti-inflammatory phenotypes. These findings suggest that reversing NF-κB signaling pathway expression can intervene in the formation of LPS-induced innate immune memory in microglia [100]. In another polysaccharide-based therapy for LPS-induced neuroinflammation, modulation of the NF-κB signaling pathway and downregulation of extracellular signal-regulated kinase (ERK) altered microglial activation and suppressed pro-inflammatory factor expression [101]. An increasing number of studies have demonstrated that persistent neuroinflammatory responses triggered by microglial activation are associated with the onset of various diseases. Targeting NF-κB to intervene in neuroinflammation provides novel miRNA targets, specifically manifested as follows: Upregulation of TNF-α induces miR-342, which directly inhibits endogenous NF-κB suppressors, leading to sustained NF-κB activation. This transition shifts microglia from a resting state to a pro-inflammatory trained state, further promoting NF-κB secretion. These findings establish the miR-342/BAG-1/NF-κB axis as a critical pathway regulating microglial immune training [102].

### 7.4. JAK-STAT Signaling Pathway

The JAK-STAT pathway is a transmembrane inflammatory signaling mechanism that rapidly transmits external signals to the nucleus upon stimulation by cytokines or interferons, thereby regulating host cell proliferation, differentiation, and survival [103]. Neuroinflammatory responses are primarily mediated by abnormal microglia activation and are regulated through the JAK-STAT pathway, which serves as the main signaling route for cytokines to IIM and coordinate adaptive immune mechanisms [104]. In multiple sclerosis (MS) induced by excessive microglial activation, high-dose CIG treatment suppresses the phosphorylation of JAK2/STAT1/STAT3, inhibits STAT1/3 nuclear translocation, and reduces promoter binding to pro-inflammatory genes. In vitro, when microglia are induced into an immune-trained state using LPS/IFN-γ, CLG treatment similarly inhibits pathway activation and alleviates neuroinflammatory responses [105]. Thus, the JAK/STAT signaling pathway represents a critical target for pharmacological inhibition of inflammatory responses. Abnormal activation of this signaling pathway serves as a core mechanism underlying the pathogenesis of numerous diseases. Studies have demonstrated that the JAK-STAT pathway is closely associated with the pathogenesis of various autoimmune disorders. For instance, in systemic lupus erythematosus (SLE), IL-6 can activate STAT3, upregulate Th17 cell differentiation, exacerbate immune responses and tissue damage, and promote the formation of immune memory [106]. Following spinal cord injury (temporary or permanent), the JAK/STAT signaling pathway becomes activated, leading to proliferation of inflammatory cells and secretion of inflammatory factors. The use of the JAK selective inhibitor tofacitinib (TOF) can reduce phosphorylation levels of STAT1 and STAT3, thereby decreasing the expression of pro-inflammatory factors such as iNOS, IL-6, TNF-α, and IL-1β. In vitro experiments have demonstrated that when HT22 cells are co-cultured with LPS-induced BV-2 cells, HT22 cells exhibit neuronal atrophy and dendritic retraction. However, after TOF inhibits JAK-STAT signaling pathway activation prior to co-culture, some neurons regain normal morphology and demonstrate neuroprotective effects [107].

In conclusion, the JAK-STAT signaling pathway is closely associated with microglia activation. Targeted inhibition of the pathway can effectively alleviate neuroinflammation, restore neuronal morphology, and exert neuroprotective effects, providing an important therapeutic strategy for immune-related diseases and neurological disorders.

## 8. Research Progress on IIM of Microglia in CNS Diseases

**Figure 2:**
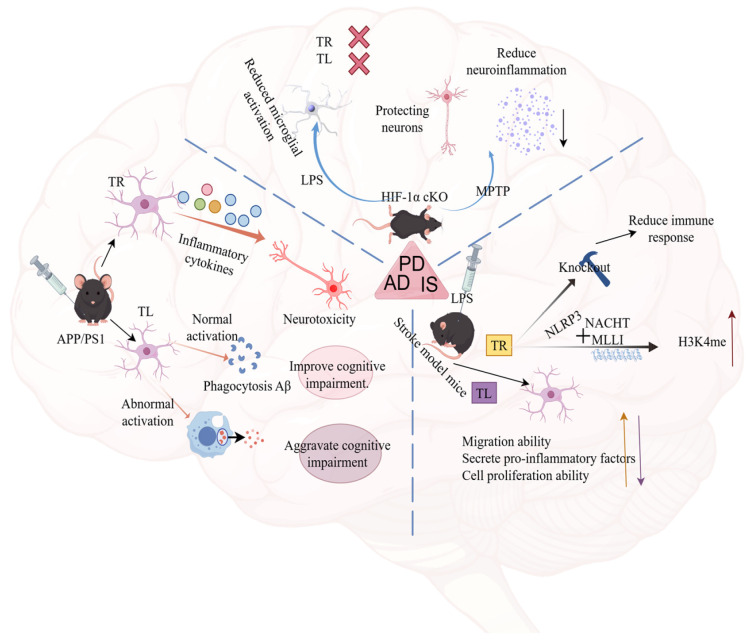
Mechanisms of microglial immunotherapy and immune tolerance in AD, PD, and IS disorders. Mechanisms underlying microglial regulation in Alzheimer’s disease (AD), Parkinson’s disease (PD) and ischemic stroke (IS) under immune training (TR) and immune tolerance (TL) states. (The image was drawn on Figdraw; https://www.figdraw.com/static/index.html#/; accessed on 19 December 2025). MPTP: 1-Methyl-4-phenyl-1,2,3,6-tetrahydropyridine, NLRP3: NOD-like receptor thermal protein domain associated protein 3, NACHT/MLLI: structural domain. Upward and downward arrows indicate increased or decreased expression of the factor, respectively. Other directional arrows represent subsequent progression. The ‘×’ symbol indicates that the TR and TL processes are inhibited.

**Figure 3:**
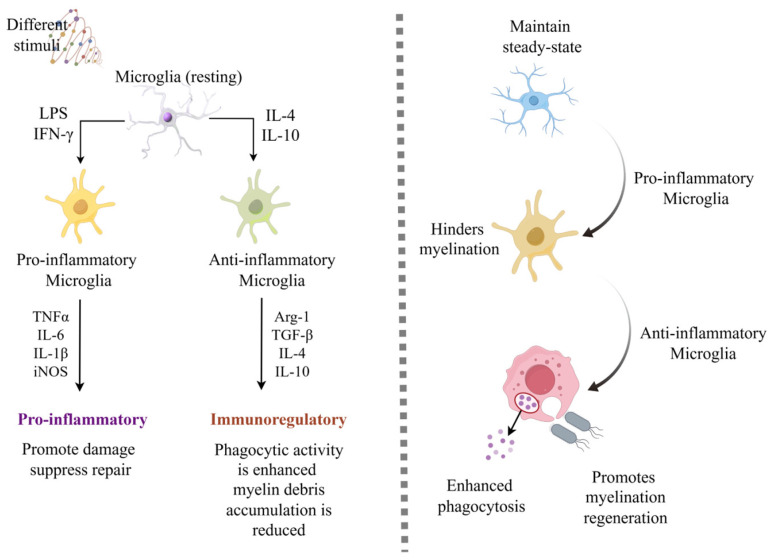
Illustration of microglial innate immune memory and microglial status in multiple sclerosis disease. Microglia exhibit MI and M2 types when encountering different stimuli, which is similar to the phenotype in multiple sclerosis diseases, indicating that the innate immune memory of microglia is a key link between aging, inflammation, and myelin repair (The image was drawn on Figdraw; https://www.figdraw.com/static/index.html#/; accessed on 7 April 2025). iNOS: Inducible Nitric Oxide Synthase, IL-10: Interleukin-10, IL-4: Interleukin-4, Arg-1: Arginase-1, TGF-β: Transforming Growth Factor Beta.

**Figure 4:**
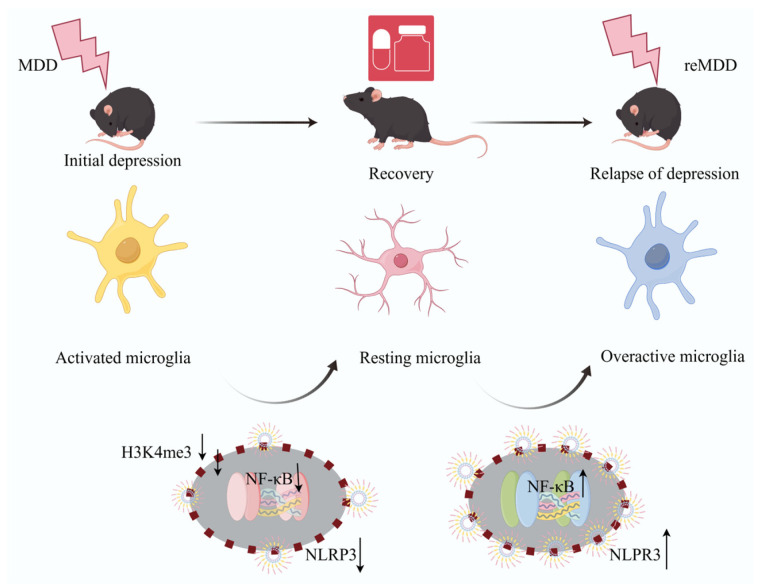
Mechanisms underlying exacerbated pathological features during recurrent depressive disorder. Microglia become activated following initial depressive episodes, then return to a quiescent state upon therapeutic recovery. Upon restimulation during disease recurrence, microglia undergo excessive activation, with NF-κB signaling promoting NLRP3 inflammasome assembly and secretion, thereby further aggravating disease pathology (The image was drawn on Figdraw; https://www.figdraw.com/static/index.html#/; accessed on 7 April 2025).

The CNS consists of the brain, brainstem, and spinal cord, serving as the core component of the nervous system [108]. CNS disorders such as AD, PD, IS and depression have become major global health challenges [109]. According to the World Health Organization (WHO), over 20% of known diseases worldwide are associated with the CNS, and the incidence rate continues to rise [110]. Glial cells constitute another critical cell population in the CNS, accounting for approximately half of all CNS cells. This group includes oligodendrocytes, microglia, and astrocytes [111]. Microglia play a pivotal role in neuroinflammatory responses and immune responses during central nervous system diseases. Through metabolic reprogramming and epigenetic alterations, they can form long-term immune memory following initial immune stimulation. This immune memory enables microglia to mount stronger responses upon re-exposure to immune attacks. As our understanding of microglial immune memory mechanisms deepens, this mechanism may provide novel therapeutic targets for disease treatment. Figure 2 summarizes the pathogenic mechanisms of IIM in AD, PD, and IS. Figure 3 and Figure 4 summarize the pathogeneses of multiple sclerosis and depression, respectively.

### 8.1. AD

AD is a neurodegenerative disorder characterized by pathological features including Aβ deposition, accumulation of hyperphosphorylated Tau protein within neurons, and intracellular neurofibrillary tangles [112]. Recent studies indicate that neuroinflammation accompanies the progression of AD and is closely associated with Aβ formation, which is considered a key factor in AD pathogenesis [113]. Indeed, neuroinflammation is now recognized as the third major pathological feature of AD, following Aβ accumulation and neurofibrillary tangles. Microglia play a pivotal role in the pathogenesis of neuroinflammation [114,115]. In AD research, intraperitoneal injection of LPS once or four times was found to induce two distinct IIM states in microglia within the brain: an immune-trained state and an immune-tolerant state. Experimental results demonstrated that a single LPS injection altered the metabolic state of the mouse brain, specifically manifested as upregulation of HIF-1α/mTOR expression, increased expression of pro-inflammatory factors, and reduced Aβ clearance capacity, thereby exacerbating deposition and leading to elevated neuronal mortality. After four consecutive days of LPS injection, pathological features of AD were improved, and behavioral studies showed enhanced cognitive function in mice [9]. In another study, primary microglial cells were treated with Aβ or LPS for 24 h. Aβ-treated microglia exhibited identical activation morphology and pro-inflammatory factor secretion patterns as those induced by LPS treatment, indicating that Aβ can also serve as an inducer for immune training in microglial cells. Additionally, metabolic alterations were observed in immune-trained cells, characterized by increased glycolytic pathway activity accompanied by suppressed oxidative phosphorylation. Since these findings confirmed that single Aβ exposure could induce immune training, researchers further utilized Aβ to induce immune tolerance in microglial cells. Notably, Aβ-induced immune tolerance was associated with metabolic dysfunction in microglial cells, contradicting previous studies suggesting immune tolerance may ameliorate disease pathology. This difference may be attributed to the inherent inducible effects of Aβ as a pathological biomarker [90]. In most studies on neuroinflammation-driven AD, pro-inflammatory microglia typically enter an immune-trained state following LPS stimulation, leading to upregulated expression of NADPH oxidase and iNOS, which produce ROS and nitric oxide (NO), resulting in neurotoxicity and exacerbation of neuroinflammation [116].

The IIM of microglia exhibits a double-edged sword effect in AD: it may both improve pathological conditions and exacerbate disease progression. This finding provides novel insights for therapeutic strategies, necessitating evaluation of patients’ microglial memory status to avoid aggravating damage solely by stimulating tolerant cells.

### 8.2. PD

PD is the second most prevalent neurodegenerative disorder worldwide. Its global incidence is increasing annually and rises with age, posing a significant public health threat [117]. The primary pathological features of PD include degeneration of dopaminergic neurons in the substantia nigra (SN) and abnormal accumulation of α-synuclein in the Lewy bodies [118]. In PD research, intraperitoneal injection of LPS twice or four times induces the generation of innate immune memory in microglia. Behavioral tests and related molecular assays revealed that administering MPTP to establish a Parkinson’s mouse model after immunological training leads to increased activation of microglia in the brain and exacerbates cognitive dysfunction. However, mice with established Parkinson’s models after immune tolerance demonstrate reversal of this phenomenon and improvement in disease pathology. Another significant finding is the critical role of HIF-1α in the formation of IIM in microglia. Gene knockout studies of HIF-1α in mice revealed IIM formation [15]. This demonstrates that HIF-1α exerts protective effects in MPTP-induced PD models by suppressing neuroinflammatory responses, indicating its critical regulatory role in both innate immune memory and MPTP-induced PD pathology. In a rat study using stereotactic LPS injection to model PD, four consecutive LPS injections were confirmed to induce peripheral immune tolerance. Compared to the control group, the immune-tolerant group exhibited significantly reduced pro-inflammatory factor expression, inhibited microglial activation, and increased survival rate of dopaminergic neurons in the substantia nigra [119]. However, other studies have demonstrated that overexpression of α-Syn impairs microglial autophagy function and promotes pathological progression of PD through Tlr4-dependent p38 and Akt-mTOR signaling pathways [120]. In the future, PD may be treated by modulating microglial autophagy.

### 8.3. Ischemic Stroke

Ischemic cell death, caused by insufficient oxygen and glucose supply, is a primary pathophysiological mechanism of post-stroke brain injury [121]. IS leads to extensive neuronal death and brain structural damage, subsequently generating pro-inflammatory molecules and cellular debris that trigger secondary inflammatory injury [122]. LPS-induced immune tolerance in mice significantly reduced post-stroke infarct volume, neuronal damage, microglial activation, and pro-inflammatory factor expression, while enhancing anti-inflammatory factor expression. In vitro experiments confirmed that LPS-induced microglial immune tolerance decreased their migratory capacity, pro-inflammatory factor expression, cell proliferation, and phagocytic function. In an established hypoxia–reperfusion model, microglia in an immune-tolerant state exhibited inhibited migration, reduced pro-inflammatory factor expression, and diminished cell proliferation, but their phagocytic capacity was significantly enhanced after hypoxia–reperfusion [123]. Cortical microinfarcts (CMIs) are relatively common in the elderly population, and patients with this condition are more prone to recurrent strokes. Feng Yiwei’s team discovered that microinfarcts can induce microglial immune training, thereby triggering pro-inflammatory responses and ischemic damage that ultimately lead to stroke. NLRP3 interacts with the MLL1 complex within microglial nuclei via its NACHT domain, elevating H3K4 methylation levels and playing a critical role in the formation of microinfarct-induced IIM. Additionally, knockout of the NLRP3 gene in microglia attenuates immune training effects and mitigates the detrimental impact of microinfarcts on recurrent strokes [124]. These findings provide potential therapeutic targets for alleviating recurrent stroke.

### 8.4. Multiple Sclerosis Disease

Multiple sclerosis (MS) is a common inflammatory disease of the CNS. Onset is predominantly between 20 and 40 years of age, with a continuously rising global incidence rate [125]. Typical pathological features include demyelination and oligodendrocyte and axonal damage [125]. Microglia play a role in continuously monitoring changes in the central nervous system microenvironment by activating defense mechanisms to provide tissue protection [96]. Microglia possess plasticity and can differentiate into pro-inflammatory or anti-inflammatory phenotypes under various environmental stimuli. Microglial activation is a key of the pathological features of MS. Upon stimulation, microglia secrete TNF-α, which induces abnormally elevated expression of vascular endothelial TRPV4. Overexpression of TRPV4 disrupts blood–brain barrier integrity, exacerbates neuroinflammation, and promotes peripheral immune cell infiltration, forming a vicious cycle that accelerates MS pathology [126]. In the autoimmune encephalomyelitis (EAE) model, as demyelination regeneration begins, microglial phenotypes transition from pro-inflammatory to anti-inflammatory [127]. This is similar to the postphenotypic changes induced by external stimuli in IIM. MS is essentially an autoimmune disease primarily targeting demyelination, with an imbalance between demyelination and repair persisting throughout the disease course [128]. The IIM state of microglia serves as a critical regulator of this imbalance. In age-related MS studies, microglial IIM undergoes abnormal remodeling. Following prolonged exposure to chronic inflammatory microenvironments, aged mice develop pathological immune memory in microglia, which exacerbates damage and inhibits repair processes. Microglia phagocytizing demyelination debris are prone to form cholesterol-overloaded foam cells, impeding demyelination regeneration. BCG-induced innate immune training can precisely target microglial IIM for epigenetic reprogramming, significantly enhancing phagocytic activity and reducing myelin fragment accumulation, thereby eliminating myelin regeneration impairment and markedly improving regenerative capacity [129].

Pathological immune memory exacerbates demyelinating damage and inhibits repair mechanisms, whereas reshaping the memory phenotype through innate immune training can break this pathological cycle. These findings provide novel therapeutic strategies for MS, with particularly significant clinical implications for elderly patients.

### 8.5. Depression

Depression is not only an emotional disorder caused by monoamine neurotransmitter imbalance but also a brain disease closely associated with dysregulation of the immune microenvironment in the CNS. Neuroinflammation plays a pivotal role in the pathogenesis and progression of depression [130,131]. Clinical data indicate that depressive-like symptoms in humans are associated with elevated levels of systemic inflammation. For instance, anxiety and depressive-like mood induced by increased interferon IFN-α expression in healthy individuals exhibit severity comparable to that observed in patients with depression [132]. Researchers found that administering two different doses of LPS via injection and analyzing blood profiles and questionnaires 24 h later revealed increased expression of pro-inflammatory factors in patients, accompanied by dose-dependent depressive mood [133]. By regulating microglial activation, changes in the brain microenvironment can be modulated. Depression is also considered a disorder associated with abnormal microglial activation [134]. Extensive evidence indicates that microglia-mediated inflammatory responses leading to alterations in immune status are closely associated with depression. Chronic stress or peripheral inflammatory stimuli increase blood–brain barrier permeability, allowing peripheral inflammatory factors to enter the CNS microglia. This activates microglial NLRP3 inflammatory bodies, resulting in dysregulation of the KYN pathway, hypothalamic–pituitary–adrenal axis dysfunction and impaired neurogenesis. These factors collectively lead to hyperexcitability of glutamatergic neurons, ultimately triggering depression [135]. It is well established that depression is a highly recurrent disorder. From an immunorelevant mechanism perspective, this may be associated with the formation of ‘traumatic memories’ by microglia in the brain. The phenomenon of symptom exacerbation upon re-exposure to stressors after patients recover their normal lives following treatment aligns with this hypothesis, which has been validated in depression model mouse experiments. Studies have found that although microglia in emotion-related brain regions have morphologically returned to a resting state, epigenetic reprogramming persists. Upon re-exposure to stressors, traumatic memories are activated, subsequently triggering the NLRP3 pathway and inducing neuroinflammatory responses, ultimately leading to depression recurrence and disease progression. Pharmacological interventions to inhibit H3K4me3 formation in microglia or downregulate NLRP3 expression have successfully prevented depression recurrence in mice. This demonstrates that preventing depression recurrence by clearing ‘traumatic memories’ from microglia is a feasible approach [136]. These findings also elucidate why severe depressive experiences increase to future episodes and provide new directions for clinical treatment. Figure 4 illustrates the mechanism of depression recurrence.

## 9. Translational and Clinical Perspectives

From a translational medicine perspective, the bidirectional regulation of immune memory through epigenetic and metabolic reprogramming has led to the emergence of several drug targets and intervention strategies with clinical translational potential. Regarding epigenetic modulators, LPS-induced innate immune training (trained immunity) exacerbates ischemic brain injury. Administration of human umbilical cord mesenchymal stem cells (hUCMSCs) reverses this detrimental immune memory by downregulating histone H3K4me1 methylation and inhibiting microglial overactivation, thereby reducing infarct volume and improving neurological function [137]. In the context of spinal cord injury, valproic acid has been shown to alleviate neuroinflammation by specifically inhibiting HDAC3, which enhances the acetylation of ATAT1 and NF-κB, prevents the formation of their pro-inflammatory transcriptional complex and reverses their nuclear transcriptional activity. This mechanism promotes the phenotypic polarization of microglia from a pro-inflammatory to an anti-inflammatory state, reduces neuroinflammation, protects the blood–spinal cord barrier, and improves motor function [138]. In a study on epigenetic reprogramming of microglial immune regulation in neuroinflammation, Huang et al. identified H3K27ac as a core epigenetic mark of microglial trained immunity. Treatment with a p300 inhibitor reduced H3K27ac levels, thereby reversing trained immunity and ameliorating neuronal damage and neuroinflammatory responses in a PD mouse model [44]. Metabolic intervention strategies also show considerable promise. During AD progression, for instance, Aβ induces metabolic reprogramming in microglia, driving them toward a highly glycolytic state with substantial lactate production. Lactate epigenetically activates PKM2 transcription via histone H4K12 lactylation, and the upregulated PKM2 further enhances glycolysis, establishing a glycolysis–H4K12la–PKM2 positive feedback loop. This loop locks microglia into a sustained pro-inflammatory phenotype, generating pathological IIM that perpetuates neuroinflammation and exacerbates neuronal and synaptic damage. Disrupting this loop through pharmacological or genetic interventions effectively reverses aberrant microglial immune memory, alleviates neuroinflammation, and improves AD-related pathology and cognitive deficits, thus offering a dual metabolic–epigenetic target for AD therapy [139]. Additionally, Aβ upregulates DGAT2 in microglia, converting excess free fatty acids (FFAs) into triglycerides (TGs) and promoting extensive lipid droplet (LD) formation. LD overload impairs microglial fatty acid oxidation (FAO), reduces Aβ phagocytosis, and solidifies a pro-inflammatory immune memory, creating a vicious cycle of “lipid droplet–phagocytic defect–inflammation–plaque.” Inhibiting DGAT2 blocks lipid droplet formation, restores metabolic homeostasis and Aβ clearance, and reverses microglial immuno-metabolic imbalance, thereby alleviating AD pathology [140]. These strategies have shown efficacy in clinical studies for reversing microglial immuno-metabolic imbalance.

Nevertheless, the clinical translation of therapies targeting human microglia still faces several major challenges. Most drugs have poor blood–brain barrier (BBB) permeability, failing to reach effective concentrations in the brain. Moreover, the high regional heterogeneity of microglia within the brain increases the risk of off-target effects when target specificity is insufficient. In summary, a thorough understanding of the epigenetic–metabolic coupling mechanisms underlying microglial immune memory, along with the development of highly specific, BBB-crossing targeted interventions, is essential for advancing this field from basic research to clinical application.

## 10. Conclusions and Outlook

Regarding CNS disorders, numerous studies have demonstrated that abnormal microglial activation exacerbates pathological responses through neuroinflammatory reactions. However, the concept of IIM in microglia has redirected research perspectives. Given that immune training and immune tolerance represent opposing mechanisms, microglia should not be regarded solely as pro-inflammatory effector cells when investigating diseases induced by microglial activation and subsequent neuroinflammation. Instead, an analytical approach incorporating metabolic reprogramming and epigenetic reprogramming of immune memory is essential. This review elucidates the concept of IIM in microglia, compares the characteristics of immune training versus immune tolerance, summarizes substances capable of inducing intrinsic immune memory in microglia, and explores the underlying mechanisms of these signaling pathways in CNS diseases.

Microglial innate immune memory acts as a double-edged sword in CNS diseases. When employing immune memory modulation therapy for such conditions, evaluating the immune memory status of microglia is crucial for achieving optimal therapeutic outcomes. Extensive studies have demonstrated that external inflammatory stimuli can induce immune memory formation; however, the intensity and duration of stimulation may paradoxically exacerbate CNS disease progression. Current research primarily relies on animal models or in vitro cell experiments, lacking direct clinical data from human brain tissues, which limits the therapeutic potential of this phenomenon in clinical neurological disorders. In-depth investigation into microglial immune memory may identify novel drug targets and provide innovative therapeutic strategies for neurodegenerative diseases, acute brain injuries, and neuroinflammatory disorders. Future studies should elucidate the causal relationship between immune memory and neurodegenerative disease progression, further clarifying the role and regulatory mechanisms of immune memory in disease pathogenesis. Targeted therapies and immunomodulatory strategies targeting microglial immune memory are expected to become pivotal directions in neurological disease treatment, opening new avenues for therapeutic advancement.

## Data Availability

No new data were created or analyzed in this study. Data sharing is not applicable to this article.

## Abbreviations

The following abbreviations are used in this manuscript:

Aβ	Amyloid β-protein
Arg-1	Arginase-1
HIF-1α	Hypoxia-Inducible Factor-1
iNOS	Inducible Nitric Oxide Synthase
IL-1β	Interleukin-1 beta
IL-4	Interleukin-4
IL-6	Interleukin-6
IL-10	Interleukin-10
JAK	Janus Kinase
LDHA	Lactate Dehydrogenase A
LDHB	Lactate Dehydrogenase B
LPS	Lipopolysaccharide
MLLI	Structural Domain
MPTP	1-Methyl-4-phenyl-1,2,3,6-tetrahydropyridine
mTOR	mechanistic Target of Rapamycin
NACHT	Structural Domain
NF-κB	Nuclear Factor-κB
NLRP3	NOD-like Receptor Thermal Protein Domain-Associated Protein 3
NOD2	Nucleotide-binding Oligomerization Domain 2
PI3K	Phosphatidylinositol-3 kinase
PKM2	Pyruvate Kinase M2
STAT	Signal Transducer and Activator of Transcription
TGF-β	Transforming Growth Factor Beta
TNF-α	Tumor Necrosis Factor-alpha
